# Polymorphisms in Long Noncoding RNA *H19* Contribute to the Protective Effects of Coal Workers’ Pneumoconiosis in a Chinese Population

**DOI:** 10.3390/ijerph13090903

**Published:** 2016-09-12

**Authors:** Qiuyun Wu, Weiwen Yan, Ruhui Han, Jingjin Yang, Jiali Yuan, Xiaoming Ji, Yi Liu, Chunhui Ni

**Affiliations:** 1 Department of Occupational Medicine and Environmental Health, Key Laboratory of Modern Toxicology of Ministry of Education, School of Public Health, Nanjing Medical University, Nanjing 211166, China; xjwqy922@163.com (Q.W.); weiwenyan911016@163.com (W.Y.); hanruhui007@163.com (R.H.); njmujj@126.com (J.Y.); yjlgogo@sohu.com (J.Y.); jxmnjmu@163.com (X.J.); liuyinjmu@163.com (Y.L.); 2 School of Public Health, Xuzhou Medical University, Xuzhou 221004, China

**Keywords:** genetics, polymorphisms, coal workers’ pneumoconiosis, long noncoding RNA, *H19*

## Abstract

The *H19* is a kind of long noncoding RNA, which has been implicated in multiple biological functions. However, the associations between genetic variants in *H19* and susceptibility of coal workers’ pneumoconiosis (CWP) have been seldom reported. In the present study, three potential polymorphisms (rs2067051, rs217727, and rs2839702) in *H19* were genotyped in a case-control study including 703 CWP cases and 705 controls. We found that individuals with the *H19* rs2067051 CT/TT genotypes showed a decreased risk of CWP compared with those with the CC genotype (adjusted OR = 0.64, 95%CI = 0.49–0.83, *p* = 0.001). Further stratified analyses revealed that the associations between variant genotypes of rs2067051 and the risk of CWP were more prominent in subjects of non-smokers (adjusted OR = 0.55, 95%CI = 0.39–0.79, *p* = 0.001) and CWP patients with Stage I (adjusted OR = 0.63, 95%CI = 0.46–0.86, *p* = 0.004). Additionally, the protective effects of *H19* rs2067051 were also evident in coal miners both with dust exposure years <25 years (adjusted OR = 0.63, 95%CI = 0.42–0.95, *p* = 0.026) and ≥25 years (adjusted OR = 0.57, 95%CI = 0.40–0.80, *p* = 0.001). Our results indicated that rs2067051 in the *H19* gene is correlated with a deceased risk of CWP in a Chinese population, which may be a potential genetic marker for prevention and intervention of CWP. Further functional studies are warranted to validate our findings.

## 1. Introduction

Coal workers’ pneumoconiosis (CWP) is a lethal fibrotic lung disease caused by inhalation and deposition of inorganic coal mine dust in the lung [1,2]. In China, 89.66% of the reported occupational cases were attributed to pneumoconiosis in 2014, of which CWP (51.52%) and silicosis (42.69%) accounted for the majority. The dust exposure level and silica content are considered to be major risk factors in the development of CWP. However, the occupational epidemiological data showed that only a portion of coal miners develop CWP, although they have the same dust exposure experience, which suggests that genetic factors also play a key role in CWP etiology [3,4]. Therefore, the identification of new genetic factors may provide new insights into the high-risk population screening, personalized precision prevention, and intervention for CWP.

Long noncoding RNAs (lncRNAs) are defined as new regulators that have been implicated in various biological processes, including disease susceptibility [5]. The lncRNA *H19*, highly conserved on human chromosome 11p15.5, plays an important role in embryogenesis throughout fetal life and decreases in mature tissues [6]. It was reported that the overexpression of *H19* is often correlated with poor prognosis in gastric cancer [7], bladder cancer [8], and lung cancer [9]. Recently, several studies have reported that the *H19* gene regulates the process of epithelial-mesenchymal transition (EMT) in colorectal cancer and esophageal cancer [10,11] increases bladder cancer metastasis by inhibiting E-cadherin expression [8] and contributes to cardiac fibroblast proliferation and fibrosis through the repression of dual-specificity phosphatase 5 and extracellular regulated protein kinases 1/2 (DUSP5/ERK1/2) [12]. It has also been found that the expression of *H19* is markedly increased in fibrotic/cirrhotic tissues of human and mouse liver [13]. More interestingly, *H19* retains relative high expression in the lung tissue and plays a critical role in the development of lung disease, such as lung cancer, chronic obstructive pulmonary disease (COPD), and so on [14,15], so the *H19* gene may be an important player in the pathological processes of pulmonary fibrosis in CWP.

Recently, several single nucleotide polymorphisms (SNPs) in lncRNA genes and their associations with disease susceptibility have been reported [16,17]. Genetic studies have indicated that sequence variants in the *H19* gene are associated with the risk of many diseases. For example, SNP rs217727 in *H19* is correlated with the risk of coronary artery disease in a Chinese population [18]. SNP rs2839698 C>T polymorphism in *H19* exon is significantly associated with the increased risk of gastric cancer by altering gene expression levels [19]. However, to date, no data has been reported on the associations of *H19* polymorphisms with the risk of CWP. Based on the above research clues, we hypothesized that SNPs in the *H19* gene may be associated with the risk of CWP. Thus, we conducted a case-control study to genotype the three candidate SNPs in the *H19* gene (rs2067051, rs217727, and rs2839702) and investigated the associations between *H19* polymorphisms and the risk of CWP in a Chinese population. The identified SNPs may be potential genetic markers for the high-risk population screening and intervention of CWP.

## 2. Materials and Methods

### 2.1. Study Subjects

This research was conducted in accordance with the Declaration of Helsinki, and the protocol was approved by the Institutional Review Board of Nanjing Medical University (approval code NJMUER201600328). All study participants were ethnic Han Chinese without a direct family or genetic relationship and provided written informed consent to participate in this study. Briefly, we consecutively recruited 703 male CWP cases and 705 male controls from the coal mines of Xuzhou Mining Business Group Co., Ltd., Xuzhou, China, between January 2006 and December 2012. The CWP cases were diagnosed by high kilovolt chest X-rays according to China National Diagnostic Criteria for Pneumoconiosis (GBZ 70-2002), which is identical to the 1980 International Labor Organization (ILO) in the judgment of opacity profusion. The CWP cases were classified into Stage I, Stage II and Stage III based on the size, profusion, and distribution range of opacities in the chest X-rays. The chest X-rays were assessed by at least two independent physicians. The control individuals were matched to the cases based upon age (within 5 years), dust exposure period, and job type. Using a double-blind investigation method, each subject received an epidemiological questionnaire by face-to-face interviews. The questionnaire focused on general information including age, smoking status, occupational histories, respiratory symptoms, and others. After the interview, venous blood samples of 5 mL were collected from all subjects.

### 2.2. SNP Selection

SNP selection was based on the HapMap database [20] and the following criteria: (a) the minor allele frequency (MAF) should be >0.05 in the Chinese Han population; (b) *r*^2^ > 0.8, calculated based on pairwise linkage disequilibrium (LD) using Haploview version 4.0 (Cambridge, MA, USA). Only the most representative SNP was selected when multiple SNPs were observed in the same haplotype block (*r*^2^ > 0.8). Finally, three SNPs (rs2067051, rs217727, and rs2839702) in the *H19* gene were included in this study. They are all located in the exons of the *H19* gene.

### 2.3. Genotyping

Conventional phenol-chloroform methods were used to extract genomic DNA from peripheral blood lymphocytes. Genotyping analysis was performed by the TaqMan allelic discrimination assay method using a 384-well format on the ABI 7900HT Real Time PCR system (Applied Biosystems, Foster City, CA, USA) according to the manufacturer’s instructions. SDS 2.4 software (Applied Biosystems, Foster City, CA, USA) was used to read the genotyping results. The primers and probes for each SNP are available on request. To ensure the quality of the experiment, 10% of the samples were selected to repeat, and the reproducibility was 100%.

### 2.4. Statistical Analysis

Deviations of the characteristics and genotype frequencies of the three SNPs between CWP cases and controls were calculated using Student’s *t*-tests (continuous variables) and *χ*^2^ tests (categorical variables). The odds ratios (ORs) and 95% confidence intervals (CIs) were estimated to examine the correlations between different genotypes and the risk of CWP from logistic regression analyses in the variant genetic models. Multiple testing corrections were calculated using Bonferroni correction. Hardy-Weinberg equilibrium (HWE) was computed by a goodness-of-fit *χ*^2^ test. All tests were two-sided, and *p* < 0.05 was considered statistically significant. All statistical analyses were performed using SAS software package (version 9.1.3; SAS Institute, Inc., Cary, NC, USA).

## 3. Results

### 3.1. Characteristics of the Study Subjects

A total of 703 male CWP cases and 705 male controls were recruited in this study. The lifestyle and occupational characteristics of participants are presented in Table 1. No significant differences were observed in regard to age (*p* = 0.086), exposure years (*p* = 0.170), or job type (*p* = 0.703) between the CWP cases and controls. There was no significant difference in smoking status between the CWP cases and controls (*p* = 0.088). However, smoking amount (pack-years) in the CWP cases was significantly less than that of the controls (*p* < 0.001). Furthermore, 61.3% of cases were in Stage I, 29.7% in Stage II, and 9.0% in Stage III.

### 3.2. Associations between H19 Polymorphisms and the Risk of CWP

The details of the three selected SNPs in *H19* are listed in Table 2. All genotyped distributions were consistent with those expected from HWE in the controls (*p* = 0.259 for rs2067051, *p* = 0.568 for rs217727, and *p* = 0.233 for rs2839702, respectively). The minor allele frequencies (MAF) of three polymorphisms were in accordance with that reported in the HapMap database [20].

Furthermore, as shown in Table 3, the genotype frequencies of rs2067051 were different between the CWP cases and controls after the adjustment of age, exposure years, pack-years smoked, and job type. A significant protective effect of CWP was found in the CT genotype compared with the CC genotype (adjusted OR = 0.67, 95%CI = 0.51–0.86, *p* = 0.002). Individuals with the T allele had a decreased risk of CWP compared with those with the C allele (adjusted OR = 0.66, 95%CI = 0.52–0.84, *p* = 0.001). Moreover, the genotype frequencies of rs2067051 were associated with the decreased risk of CWP both in the dominant (adjusted OR = 0.65, 95%CI = 0.50–0.84, *p* = 0.001) and additive genetic model (adjusted OR = 0.65, 95%CI = 0.51–0.82, *p* = 0.001). However, no significant associations were found between the genotypes of the other two SNPs (rs217727, rs2839702) and the risk of CWP.

### 3.3. Stratified Analysis between the Genotypes of H19 rs2067051 and the Risk of CWP

The stratified analysis of the associations between rs2067051 and the risk of CWP are listed in Table 4. Individuals with the CT/TT genotypes had a significantly decreased risk of CWP compared with those with the CC genotype (adjusted OR = 0.64, 95%CI = 0.49–0.83, *p* = 0.001), particularly among subgroups of non–smokers (adjusted OR = 0.55, 95%CI = 0.39–0.79, *p* = 0.001) and CWP patients with Stage I (adjusted OR = 0.63, 95%CI = 0.46–0.86, *p* = 0.004). We also foundthat the protective effects of *H19* rs2067051 were evident in coal miners both with dust exposure years <25 years (adjusted OR = 0.63, 95%CI = 0.42–0.95, *p* = 0.026) and ≥25 years (adjusted OR = 0.57, 95%CI = 0.40–0.80, *p* = 0.001). In addition, the lncRNASNP databases [21] were used to predict the functions of rs2067051 and other variants in high LD. The results are listed in Supplementary Materials Table S1.

## 4. Discussion

CWP is a chronic fibrotic lung disease caused by occupational exposure to respirable coal mineral particles. However, with the same exposure, only some of the coal miners develop CWP during their lifetime and in addition, the lung fibrotic progression is not the same for the CWP patient. The genetic variations contribute to the individual susceptibility and the severity of CWP. Our previous research has shown that three functional SPARC SNPs (rs1059279, rs1059829, and rs1053411) are associated with the increased risk of CWP [22]. Additionally, rs522616 in the promoter region of the matrix metalloproteinase 3 (MMP3) gene is correlated with a decreased risk of CWP, especially among the subgroup of no smokers and patients with Stage I [23].

To date, evidence is accumulating that genetic variants in lncRNAs are correlated with the risk of multiply diseases [24]. *H19* is an imprinted gene transcribing a long noncoding RNA, which is significantly decreased after birth [9]. Although the exact roles remain unclear, the latest studies found that the partly clarified regulatory mechanisms of *H19* covered several important features and major genes of the fibrotic diseases [10,11,12,13], so *H19* may play a significant role in the development of CWP. However, the correlations between *H19* genetic variants and the risk of CWP have not been explored. In our previous study, we have performed genome-wide association studies (GWAS) for the risk of pneumoconiosis [3]. Since only one captured SNP in the *H19* gene in our original GWAS data was found unreliable (HWE < 0.001), we selected the three SNPs in the *H19* gene (rs2067051, rs217727, and rs2839702) by traditional candidate gene approach.

In this case-control study, we found that rs2067051 CT/TT genotypes had a significantly decreased risk of CWP compared with the CC genotype in a Chinese population. This suggested that *H19* genetic variants were significantly associated with the risk of CWP. So far as we know, this is the first report on the associations between *H19* genetic variants and the risk of CWP.

The rs2067051 is located in the exon of *H19* and is associated with a decreased risk of coronary artery disease in a Chinese population [18]. The variant genotypes of rs2067051 contribute to the risk of low birth weight in Memphis and Jackson populations [25]. Numerous studies have shown that adults born at very low birth weight are significantly associated with reduced lung function in the future [26]. Are there any direct or indirect correlations between the low birth weight and the risk of CWP? This question is very interesting, and we need to collect the birth weight data and evaluate the associations based on our cohort study in the future. Additionally, *H19* exerts key roles through diverse mechanisms. For example, *H19* acts as a competitive endogenous RNAs (ceRNAs) to regulate the let-7 family of miRNAs [27]. *H19* is also a precursor for miR-675 that enhances the aggressive phenotype of breast cancer cells [28]. However, the functional effects of this variant rs2067051 in *H19* have not been explored in the present study. Here, we used the bioinformatics databases to predict its functions and found that this variant can influence the structure of *H19*. Moreover, some SNPs of *H19* in high LD with rs2067051 can affect the interactions of *H19* with microRNAs (miRNAs). Therefore, further studies are needed to explore the functions based on these two possible mechanisms.

Our stratified analysis also revealed that those with the variant genotypes of rs2067051 had a significantly decreased risk of CWP among non-smokers. The observed differences may be partly explained by the associated effects of cigarette smoke with silicosis, which are participated in the progression of lung fibrosis [29,30]. Meanwhile, the protective effects of *H19* rs2067051 were evident in coal miners both with short and long dust exposure history. Dust exposure history is a crucial factor in the development of CWP. The reports from our research group showed that the protective effects of some SNPs in protein-coding genes were evident in coal miners with a long exposure history [31,32]. However, no significant associations were observed between the genotypes of *H19* rs2067051 and years of dust exposure in this study. The reasons for these inconsistent results are still unknown. It is possible that protein-coding genes and lncRNAs polymorphisms exert different molecular mechanisms in the pathogenesis of CWP. Moreover, we also noted that the *H19* rs2067051 CT/TT carriers had a significantly decreased risk of developing Stage I. These findings may arise due to different mechanisms regulating the different progression of CWP, and the *H19* genetic variants may affect the progression differently.

Several limitations in this study must be addressed. First, the possibility of selection bias of participants could not be avoided in this population-based case-control study. Second, our sample size is relatively moderate, and the statistical power of the study is limited. Third, this study was performed in a Chinese Han population, and our findings should be extrapolated to other regions and ethnic groups and be replicated in other independent cohorts. Fourth, the clear functions of the variant genotypes have not been explored in the present study. Therefore, further studies in larger and more diverse populations are needed to validate our results.

## 5. Conclusions

The present study indicated that *H19* SNP rs2067051 is correlated with a decreased risk of CWP in a Chinese population, which may provide a new genetic marker for the high-risk population screening and early intervention of CWP. Further functional studies are warranted to validate our findings.

## Figures and Tables

**Table 1:** Demographic and selected variables among the coal workers’ pneumoconiosis (CWP) cases and controls.

Variables	CWP Cases (*n* = 703)		Controls (*n* = 705)		*p*
	N	%	N	%	
Age, years (mean ± SD)	67.26 ± 10.78		66.32 ± 9.72		0.086
Exposure years (mean ± SD)	24.91 ± 8.54		25.48 ± 7.06		0.170
**Smoking status**					0.088
Never	374	53.2	343	48.7	
Ever	329	46.8	362	51.3	
**Pack-years smoked**					<0.001
0	374	53.2	343	48.7	
0–20	201	28.6	165	23.4	
>20	128	18.2	197	27.9	
**Job type**					0.703
Tunnel and coal mining	590	83.9	599	85.0	
Transport	40	5.7	42	6.0	
Others	73	10.4	64	9.1	
**Stage**					
I	431	61.3			
II	209	29.7			
III	63	9.0			

**Table 2:** Primary information of single nucleotide polymorphisms (SNPs) in *H19* gene.

SNPs	Region	dbSNPAllele	MAF		HWE^a^
			Case	Control	
rs2067051	Exon	C>T	0.0971	0.1368	0.259
rs217727	Exon	G>A	0.3459	0.3755	0.568
rs2839702	Exon	A>C	0.2905	0.2939	0.233

HWE^a^ (Hardy-Weinberg equilibrium) *p*-value in the control group. MAF: minor allele frequencies.

**Table 3:** Distributions of genotypes of *H19* and their associations with the risk of coal workers’ pneumoconiosis (CWP).

Variables	CWP Cases		Controls		OR (95%CI) ^a^	*p* ^a^	OR (95%CI) ^b^	*p* ^b^	*p* ^c^
	N	%	N	%					
rs2067051	*n* = 695		*n* = 691						
CC	563	81.0	511	74.0	1.00		1.00		
CT	129	18.6	171	24.7	0.68 (0.53–0.89)	0.004	0.67 (0.51–0.86)	0.002	0.048
TT	3	0.4	9	1.3	0.30 (0.08–1.12)	0.074	0.28 (0.07–1.05)	0.059	1.000
C allele	1255	90.3	1193	86.3	1.00		1.00		
T allele	135	9.7	189	13.7	0.68 (0.54–0.86)	0.001	0.66 (0.52–0.84)	0.001	0.024
Dominant					0.67 (0.52–0.86)	0.002	0.65 (0.50–0.84)	0.001	0.024
Recessive					0.33 (0.09–1.22)	0.096	0.31 (0.08–1.15)	0.080	1.000
Additive					0.67 (0.52–0.85)	0.001	0.65 (0.51–0.82)	0.001	0.024
rs217727	*n* = 691		*n* = 683						
GG	296	42.8	270	39.5	1.00		1.00		
AG	312	45.2	313	45.8	0.91 (0.72–1.14)	0.413	0.91 (0.72–1.15)	0.422	1.000
AA	83	12.0	100	14.7	0.76 (0.54–1.06)	0.103	0.74 (0.53–1.04)	0.087	1.000
G allele	904	65.4	853	62.4	1.00		1.00		
A allele	478	34.6	513	37.6	0.88 (0.75–1.03)	0.105	0.87 (0.75–1.02)	0.095	1.000
Dominant					0.87 (0.70–1.08)	0.213	0.87 (0.70–1.08)	0.210	1.000
Recessive					0.80 (0.58–1.09)	0.152	0.78 (0.57–1.07)	0.128	1.000
Additive					0.89 (0.75–1.03)	0.108	0.88 (0.75–1.02)	0.090	1.000
rs2839702	*n* = 697		*n* = 689						
AA	363	52.1	350	50.8	1.00		1.00		
CA	263	37.7	273	39.6	0.93 (0.74–1.16)	0.519	0.94 (0.75–1.18)	0.601	1.000
CC	71	10.2	66	9.6	1.04 (0.72–1.50)	0.845	1.03 (0.72–1.50)	0.839	1.000
A allele	989	70.1	973	70.6	1.00		1.00		
C allele	405	29.1	405	29.4	0.98 (0.84–1.16)	0.845	0.99 (0.84–1.17)	0.900	1.000
Dominant					0.95 (0.77–1.17)	0.633	0.96 (0.78–1.19)	0.710	1.000
Recessive					1.07 (0.75–1.52)	0.705	1.07 (0.75–1.52)	0.725	1.000
Additive					0.99 (0.84–1.15)	0.850	0.99 (0.84–1.16)	0.903	1.000

Abbreviations: Dominant: wild homozygote versus heterozygote and mutational homozygote; Recessive: wild homozygote and heterozygote versus mutational homozygote; Additive: wild homozygote versus heterozygote versus mutational homozygote; ^a^ Unadjusted for age, exposure years, pack-years smoked, and job type in logistic regression model; ^b^ Adjusted for age, exposure years, pack-years smoked, and job type in logistic regression model; ^c^ Bonferroni correction.

**Table 4:** Stratified analysis between the genotypes of *H19* rs2067051 and the risk of coal workers’ pneumoconiosis (CWP).

Variables	Cases/Controls	Genotypes (Cases/Controls)				OR (95%CI) ^a^	*p* ^a^
		CC		CT/TT			
		N	%	N	%		
Total	695/691	563/511	81.0/74.0	132/180	19.0/26.0	0.64 (0.49–0.83)	0.001
**Exposure Years**							
<25	315/322	256/246	81.3/76.4	59/76	18.7/23.6	0.63 (0.42–0.95)	0.026
≥25	380/369	307/265	80.8/71.8	73/104	19.2/28.2	0.57 (0.40–0.80)	0.001
**Smoking Status**							
Never	368/335	300/240	81.5/71.6	68/95	18.5/28.4	0.55 (0.39–0.79)	0.001
Ever	327/356	263/271	80.4/76.1	64/85	19.6/23.9	0.75 (0.51–1.10)	0.138
**Pack-Years Smoked**							
0	368/335	300/240	81.5/71.6	68/95	18.5/28.4	0.55 (0.39–0.79)	0.001
>0–20	199/163	158/123	79.4/75.5	41/40	20.6/24.5	0.76 (0.46–1.27)	0.297
>20	128/193	105/148	82.0/76.7	23/45	18.0/23.3	0.70 (0.39–1.25)	0.223
**Stage**							
I	426/691	352/511	82.6/74.0	74/180	17.4/26.0	0.63 (0.46–0.86)	0.004
II	207/691	160/511	77.3/74.0	47/180	22.7/26.0	0.69 (0.47–1.02)	0.061
III	62/691	51/511	82.3/74.0	11/180	17.7/26.0	0.51 (0.25–1.03)	0.060

^a^ Adjusted for age, exposure years, pack-years smoked, and job type in logistic regression model.

## Supplementary Materials

The following are available online at www.mdpi.com/1660-4601/13/9/903/s1, Table S1: Functions prediction of rs2067051 and genetic variants in high linkage disequilibrium (LD).
