# Supplementary material for: Polymorphisms in Long Noncoding RNA H19 Contribute to the Protective Effects of Coal Workers’ Pneumoconiosis in a Chinese Population
Source: Int J Environ Res Public Health. 2016 Sep 12;13(9):903. doi: 10.3390/ijerph13090903 (PMC5036736; doi:10.3390/ijerph13090903)
Supplement: Supplementary file 1 [file ijerph-13-00903-s001.pdf]

Supplementary Materials: Polymorphisms in Long Noncoding RNA *H19* Contribute to the Protective Effects of Coal Workers' Pneumoconiosis in a Chinese Population

Qiuyun Wu, Weiwen Yan, Ruhui Han, Jingjin Yang, Jiali Yuan, Xiaoming Ji, Yi Liu and Chunhui Ni

Supplementary Table S1

Table S1. Functions prediction of rs2067051 and genetic variants in high linkage disequilibrium (LD).

SNPs	r^2	Ref>Alt	miRNA:lncRNA	Structure
rs2067051	1.00	C>T	×	√
rs3741219	0.91	A>G	√	√
rs10840159	0.91	A>G	√	√
rs2839701	0.90	C>G	√	√
rs2075745	0.98	A>G	√	√
rs2075744	0.98	A>G	√	√
rs2839698	0.97	G>A	√	√

© 2016 by the authors.Submitted for possible open access publication under the terms and conditions of the Creative Commons Attribution (CC-BY) license (http://creativecommons.org/licenses/by/4.0/).
